# *Morus alba* L. Leaves – Integration of Their Transcriptome and Metabolomics Dataset: Investigating Potential Genes Involved in Flavonoid Biosynthesis at Different Harvest Times

**DOI:** 10.3389/fpls.2021.736332

**Published:** 2021-11-16

**Authors:** Ding-Qiao Xu, Shu-Yan Cheng, Jun-Qing Zhang, Han-Feng Lin, Yan-Yan Chen, Shi-Jun Yue, Meng Tian, Yu-Ping Tang, Yu-Cheng Zhao

**Affiliations:** ^1^Key Laboratory of Shaanxi Administration of Traditional Chinese Medicine for TCM Compatibility, School of Pharmacy, Shaanxi University of Chinese Medicine, Xi’an, China; ^2^Department of Resources Science of Traditional Chinese Medicines, State Key Laboratory of Natural Medicines, School of Traditional Chinese Pharmacy, China Pharmaceutical University, Nanjing, China; ^3^Department of Metabolism, Digestion and Reproduction, Faculty of Medicine, Imperial College London, London, United Kingdom

**Keywords:** *Morus alba* L., transcriptome, metabolomics, harvest time, flavonoid biosynthesis

## Abstract

The mulberry leaf is a classic herb commonly used in traditional Chinese medicine. It has also been used as animal feed for livestock and its fruits have been made into a variety of food products. Traditionally, mulberry (*Morus alba* L.) leaf harvesting after frost is thought to have better medicinal properties, but the underlying mechanism remains largely unsolved. To elucidate the biological basis of mulberry leaves after frost, we first explored the content changes of various compounds in mulberry leaves at different harvest times. Significant enrichment of flavonoids was observed with a total of 224 differential metabolites after frost. Subsequently, we analyzed the transcriptomic data of mulberry leaves collected at different harvest times and successfully annotated 22,939 unigenes containing 1,695 new genes. Kyoto Encyclopedia of Genes and Genomes (KEGG) analysis revealed 26, 20, and 59 unigenes related to flavonoids synthesis in three different groups harvested at different times. We found that the expression levels of flavonoid biosynthesis-related unigenes also increased when harvested at a delayed time, which was consistent with the flavonoid accumulation discovered by the metabolomic analysis. The results indicated that low temperature may be a key trigger in flavonoid biosynthesis of mulberry leaves by increasing the expression of flavonoid biosynthesis-related genes. This study also provided a theoretical basis for the optimal harvest time of mulberry leaves.

## Introduction

Mulberry leaves, the dried leaves of *Morus alba* L. which belongs to the family *Moraceae* and genus *Morus* (Wei, 2015), are among the most used traditional Chinese medicine material (Yang et al., 2010). Moreover, it is also an excellent source of functional nutraceutical food. Mulberry leaves contain carbohydrates, amino acids, fatty acids, and other bioactive compounds with good anti-oxidation, antibacterial, anti-inflammatory, anti-hypoglycemic, and anti-aging effects (Srivastava et al., 2006; Cho et al., 2007; Hunyadi et al., 2012; Park et al., 2013; Wang et al., 2015; Mahboubi, 2019). Several value-added products are developed from mulberry leaves such as mulberry tea, salads, and supplement capsules. Mulberry leaf has been successfully used as a medicinal and edible resource for over 4,000 years. Traditional Chinese pharmacists have always believed that the pharmacological effect of medicinal herbs is closely related to harvest time. Chinese pharmacopeia also suggested that it would be better to harvest the mulberry leaves after than before the winter frost (Commission, 2020), which may improve their pharmacological effects. The study of Zhang et al. (2015) found that after frost, mulberry leaves have the effects of resolving exterior with pungent and cool natured drugs, which are weakened to a certain extent when harvested before frost. However, there are few reports that focus on its underlying mechanisms.

It is generally accepted that biosynthesis and accumulation of plant secondary metabolites are largely influenced by various environmental factors (Li et al., 2020). In general, genetic background determines the secondary metabolite profile of species, whereas environmental factors can cause prominent qualitative and quantitative changes to the metabolite composition. For instance, a previous study concluded that *Fragaria vesca* L. grew in natural habitats contained significantly more flavonoids and phenolic acids in its fruits, compared with those harvested from cultivation (Najda et al., 2014). The study of Jochum et al. (2007) studied the temperature effect on the saponin content of *Panax quinquefolium*, and the results showed that the saponin content of *P. quinquefolium* root significantly increased when the temperature increased. The research of Wang on *Ginkgo biloba* also showed that low temperature and moist conditions induced the expression of key enzymes in flavonoid biosynthesis in *G. biloba* leaves, leading to an increasing of flavonoid contents (Wang et al., 2013). Thus, we speculated that temperature change would be an important factor affecting secondary metabolites in mulberry leaves.

Previous, studies have shown that the mulberry leaves are rich in flavonoids (Wei, 2015; Chan et al., 2016), which account for up to 1–3% of the dry weight of mulberry leaves (Yang et al., 2003). Flavonoids form a group of phenolic secondary metabolites ubiquitously present in higher plants (Zhao et al., 2015). The basic structure of flavonoids is 2-phenyl-benzo[α]pyrane, which consists of fifteen carbon atoms arranged in three rings (C6-C3-C6) (Nijveldt et al., 2001). Due to the different patterns of the substitution of the ring, there are many derivatives, such as isoflavones, flavonols, flavanones, and chalcones (Park et al., 2013; Wei, 2015). Flavonoids are produced by the phenylpropanoid metabolic pathway, which is a common metabolic pathway in plants (Saito et al., 2013; Tohge et al., 2017). Phenylalanine ammonia-lyase (PAL) is the first enzyme in the phenylpropionate pathway, which catalyzes the non-oxidative deamination of phenylalanine to *trans-*cinnamic acid (Fang et al., 2005). Cinnamate 4-hydroxylase (C4H) then converts cinnamic acid to p-coumaric acid, which is then converted by 4-coumaroyl-CoA ligase (4CL) enzyme to 4-coumaroyl coenzyme A and, finally, leads different subgroups by enzymatic catalysis (Rodriguez et al., 2017). Among subgroups, chalcone is an important flavonoid class, which is produced by chalcone synthase (CHS). Some other important enzymes also have vital effects in this pathway. For instance, molecular characterization of isoliquiritigenin 3′-dimethylallyltransferase (MaIDT) in mulberry leaves has been reported, and the results showed that MaIDT might be used for the regiospecific prenylation of flavonoids to produce bioactive compounds (Wang et al., 2014). Furthermore, the decreased temperature has also been confirmed to elevate the flavonoid accumulation in plants. The total accumulation of flavonoids (genistein, daidzein, and genistein) in soybean (*Glycine max*) roots was increased after being treated at a low temperature for 24 h (Janas et al., 2002). Low temperature-induced anthocyanin accumulation in leaves and stems of *Arabidopsis thaliana* and facilitated anthocyanin synthesis through the phenylpropanoid pathway associated with increased transcripts of flavonoid biosynthetic genes including PAL and CHS (Leyva et al., 1995). The expression of the UDP-glucose flavonoid 3-O-glucosyltransferase (UFGT) in mulberry leaves could be induced by low temperature and resulted in the accumulation of flavonoid glycosides (Yu et al., 2017). Thus, we hypothesized that flavonoid synthesis in mulberry leaves increases under conditions of low temperature, as a stress response to resist chilling.

Metabolomics is defined as the study of the complete set of metabolites synthesized by an organism in response to genetic or environmental changes, aiming to provide a link between genotypes and phenotypes (Fiehn, 2002). Transcriptomics can be used to analyze the differences in gene expression levels of medicinal plants under abiotic pressure, which lays the foundation for the regulation of plant secondary metabolism (Patra et al., 2013). By combining the two kinds of analyses strategy, the difference of metabolites in different groups, as well as their gene expression level, can be illustrated. For example, the molecular mechanism of anthocyanin accumulation in *Solanum melongena* L. was determined by combined transcriptome and metabolome analysis (Zhang et al., 2014). Integrated analyses were also used in the studies of the biosynthesis pathway of the podophyllotoxin in *Podophyllum hexandrum* (Lau and Sattely, 2015). The results revealed that flavonoids were the main differential accumulative metabolites (mDAMs), and their contents increased with the temperature decrease. In this study, using metabolomics and transcriptomics analysis, we proved the importance of temperature in the regulation of secondary metabolites production and its relationship with the quality of medicinal plants. This paper also provided guidance for the preference of harvesting time of mulberry leaves. Additionally, considering that mulberry leaf constitutes a functional food and medicinal plant commercialized for the treatment of hyperlipidemia, the identification of low temperature modulating the biosynthesis of these metabolites could benefit in the development of reliable commercial products.

## Materials and Methods

### Plant Material and Grouping Omics Analyzed Samples

**FIGURE 1:**
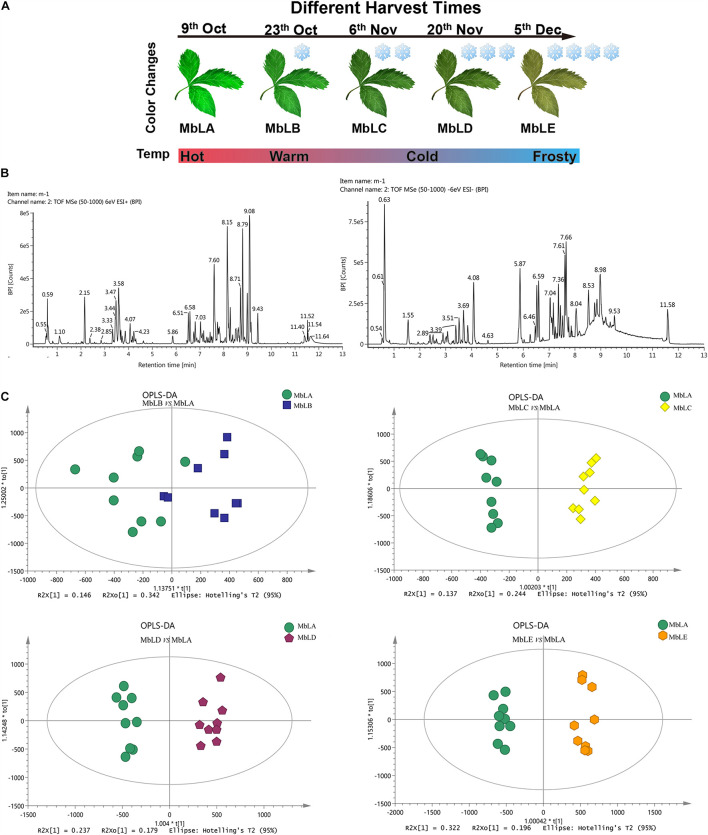
Metabolite accumulation in the mulberry leaves at five periods. **(A)** Mulberry leaves picked on October 6th were recorded as MbLA group; those picked on October 23th were recorded as MbLB group; those picked on November 6th were recorded as MbLC group; those picked on November 20th as MbLD group, and those picked on December 5th were recorded as MbLE group. **(B)** Analysis of compounds extracted from the sample of mulberry leaves using UPLC-Q-TOF/MS. Base peak intensity (BPI) chromatogram of extract analyzed by UPLC-Q-TOF-MS in positive and negative ion mode. **(C)** Score plots for OPLS-DA analysis based on UPLC-Q-TOF-MS data of mulberry leaves extracts obtained from the MbLB to MbLE, with MbLA as the control group. Each point represents an independent biological replicate, and each ellipse represents the 95% CI.

Fresh mulberry leaves were picked from mulberry trees [located in Hanzhong City (106.21°E, 32.53°N), Shaanxi Province, China] at different times. From mulberry leaves A (MbLA) to E (MbLE), the temperature gradually dropped as there was a delay in harvesting time (on October 9th, 23rd, November 6th, 20th, and December 5th in the Beijing time zone and the average temperature are 20, 13, 10, 6, and 4°C, respectively) (Figure 1A). The descent of the frost was on October 23rd. Each leaf was split into two equal parts along with the midvein: one-half was used for metabolomic analysis, and the other half was used for transcriptomic analysis. For transcriptomic analysis, three biological replicas were conducted, whereas for metabolomics analysis, additional six biological replicas were needed which up to nine replicas. All materials were immediately frozen in liquid nitrogen to prevent RNA degradation.

### Sample Extraction

For the metabolomics experiments, every nine samples from each group were used for separate analysis. Then, 60 mg of leave samples were weighed, and 20 μL of 2-chloro-l-phenylalanine (0.3 mg/ml, dissolved in methanol as internal standard) and 0.6 ml of mixed solution [methanol/water = 7/3 (*v:v*)] were added. The samples were homogenized for 2 min and were extracted for 30 min by sonication. They were then placed at −20°C for 20 min and centrifuged at 13,000 × *g* for 15 min. Afterward, 100 μL supernatant from each tube was collected, filtered through 0.22 μm microfilters, and transferred to liquid chromatography (LC) vials. Quality control (QC) samples were prepared by mixing aliquots of all samples to be a pooled sample and then analyzed using the same method with the analytic samples. The QCs were injected at regular intervals (every 10 samples) throughout the analytical run to provide a data set from which repeatability could be assessed.

### Metabolite Profiling Using Ultra-High-Performance Liquid Chromatography-Quadrupole Time-of-Flight Mass Spectrometry

Analyses were performed using a Waters UPLC I-class system equipped with a binary solvent delivery manager and a sample manager, coupled with a Waters VION IMS Q-TOF Mass Spectrometer equipped with an electrospray ionization source (Waters Corporation, Milford, MA, United States). Samples were analyzed using an ACQUITY UPLC BEH C18 column (2.1 mm × 100 mm, 1.7 μm, Waters Corporation, Milford, MA, United States) in positive and negative modes. The column temperature was maintained at 45°C and the flow rate of the mobile phase was 0.4 ml/min, accompanied by an injection volume of 3 μL. Mobile phase A was aqueous formic acid [0.1% (*v/v*) formic acid], while mobile phase B was acetonitrile [0.1% (*v/v*) formic acid]. The separation was achieved using the following gradient: 5–20% B over 0–2 min, 20–60% B over 2–8 min, and 60–100% B over 8–12 min. The composition was held at 100% B for 2 min, then 14–14.5 min, 100% to 5% B, and 14.5–15.5 min holding at 5% B. The automatic sampler was set at 4°C during the analysis of all samples.

All data were collected in MS^*E*^ mode, and the parameters were as follows: Capillary voltage was 3 kV for positive mode. Source temperature was set at 150°C with a cone gas flow of 50 L/h, and desolvation temperature was set at 500°C with a desolvation gas flow of 900 L/h. Leucine enkephalin (Waters Co., Manchester, United Kingdom) was used as the lock mass generating a reference ion at *m/z* 556.2771 in positive mode or *m/z* 554.2615 in negative mode, which was introduced by a lock spray at 5 μL/min for data calibration. The MS^*E*^ data were acquired in centroid mode using ramp collision energy in two scan functions. For Function 1 (low energy), scan range 50–1,000 Da, scan time 0.25 s, and collision energy 10 V were set. In the case of Function 2 (high energy), scan range 50–1,000 Da, scan time 0.25 s, and a collision energy ramp 20–50 V were employed.

### Data Processing and Analysis of Metabolites

The UPLC-Q-TOF/MS raw data were imported into Progenesis QI V2 (Waters Corporation, Milford, MA, United States) to the clean background noise, be normalized by a reference sample, correct the retention time, pick the peak, and identify compounds with databases such as METLIN, HMDB, and Lipid Maps. The resulting matrix was further reduced by removing any peaks with missing values (ion intensity = 0) in more than 60% of samples. The internal standard was used for data QC (reproducibility). The positive and negative data were combined to get a combined data set, which was imported into SIMCA-P^+^14.0 software (Umetrics, Umeå, Sweden). Principle component analysis (PCA) and (orthogonal) partial least-squares-discriminant analysis (O) PLS-DA were performed to visualize the metabolic alterations among experimental groups, after mean centering and unit variance scaling. OPLS-DA concentrated group discrimination in the X block related to Y into the first component, with the remaining unrelated variations orthogonal to Y in subsequent components. MS data of the second independent experiment of leaves were used as the test data to objectively assess R^2^, Q^2^, and misclassification rate of established models based on permutation test (2,000 times) that was performed to further validate the supervised model. The significant different metabolites were determined based on the combination of a statistically significant threshold of variable importance in the projection (VIP) values obtained from the OPLS-DA model and two-tailed Student’s *t*-test (*p*-value) on the raw data, and the metabolites with VIP values larger than 1 and *p*-values <0.05 were considered significantly different between the compared groups.

### Total RNA Extraction

For every three samples at different harvest times, the total RNA of mulberry leave samples was isolated once using TransZol Plant reagent (TransGen Biotech, Beijing, China) according to the recommendations of the manufacturer. The quantity and quality of RNA were determined using a SpectraMax Plus 384 spectrophotometer (Molecular Devices, Sunnyvale, CA, United States) and 1% agarose gels. In addition, the quantity and quality of RNA were determined by Agilent 2100 Bioanalyzer system (Agilent Technologies, Santa Clara, CA, United States) and the one representative result is listed in Supplementary Figure 1. The RIN, 28S/18S, OD260/280, and OD260/OD230 ratios of all samples are listed in Supplementary Table 3. All samples were treated with DNase I (Takara, Dalian, China) at a concentration of 1 unit/μg of total RNA for 30 min to remove the potential DNA.

### cDNA Library Construction and Transcriptome Sequencing

A cDNA library was prepared with a kit provided by Illumina according to the recommendations of the manufacturer and previously used methods (Yuan et al., 2015). Then, poly (A) mRNA was purified from the total RNA using oligo(dT) beads. After purification, mRNA was sheared into small pieces using fragmentation buffer. The first-strand cDNA was annealed with random primers using cleaved mRNA fragments as templates. The second-strand cDNA was synthesized with DNA polymerase I and RNase H. Subsequently, the cDNA fragments were purified and ligated to index adapters. Finally, the cDNA library was constructed and subjected to Illumina HiSeq 2500 system for high-throughput sequencing. The raw data were converted into fastaq format and then compressed as.gz files to be transferred to the National Center for Biotechnology Information (NCBI). The Sequence Read Achieve (SRA) sequence database under project accession number was PRJNA533997.

Due to the error rate in the raw data, low-quality-sequence fragments were removed *via* slip-window sampling using the following parameters: quality threshold of 20 (error rate = 1%), a window size of 5 bp, and length threshold of 35 bp. To adjust the pollution of the reads, 10^5^ sequences were randomly selected for sequence alignment of the nr reads at an *E*-value of <1e^–10^ and a coverage level of >80%. After the pollution of the reads being cleaned, the good reads were used to assemble transcripts and unigenes using Trinity software (version trinityrnaseq_r2013-02-25)^1^. The unigenes representing the longest transcripts at each locus were assembled using the Chrysalis cluster module in Trinity program. To normalize the abundance of the transcripts, a *k*-mer value of 25 reads per kilobase per million mapped reads (RPKM) (Wagner et al., 2012) was applied and defined in this way:


(1) $$\text{RPKM}=\frac{\mathrm{total}\,\mathrm{exon}\,\mathrm{read}}{\mathrm{mapped}\,\mathrm{reads}\,\left(\text{millions}\right)*\mathrm{exon}\,\mathrm{length}\,\left(\text{KB}\right)}$$

### Functional Annotation and Classification

To find the most descriptive annotation for each transcript sequence, The Basic Local Alignment Search Tool (BLAST) searches (Altschul et al., 1997) were conducted based on sequence similarities using a series of databases (Kanehisa et al., 2002; Dimmer et al., 2012), with the significance threshold set at an *e*-value of ≤1e^–5^. The functional categories of these unique sequences were analyzed using the Gene Ontology (GO)^2^ database, AGI codes and the TAIR GO slim program were provided by TAIR (Lamesch et al., 2012). Pathway assignments were conducted based on the Kyoto Encyclopedia of Genes and Genomes (KEGG) mapping results, and enzyme commission (EC) numbers were assigned to the unique sequences (Aoki-Kinoshita and Kanehisa, 2007). The KOG/COGs (clusters of orthologous groups) of the proteins were aligned to the entries in the EggNOG database to predict and classify the possible functions of the unigene products^3^.

### Integrative Analysis of Metabolomics and Transcriptome

Metabolites and differentially expressed genes (DEGs) involved in flavonoid biosynthesis and metabolism in KEGG pathways were selected for integrative analysis. Metabolites used for correlation analysis were filtered according to VIP > 1, *p*-value <0.05, and | Log_2_^Fold Change^| ≥ 2. Pearson correlation coefficients and *p*-values were calculated for metabolomics and transcriptome data integration using the Spearman method (Kyoungwon et al., 2016).

## Results

### Metabolomics Analysis Revealed Abundant and Diverse Flavonoid Enrichment in Mulberry Leaves Under Cold Stress

**FIGURE 2:**
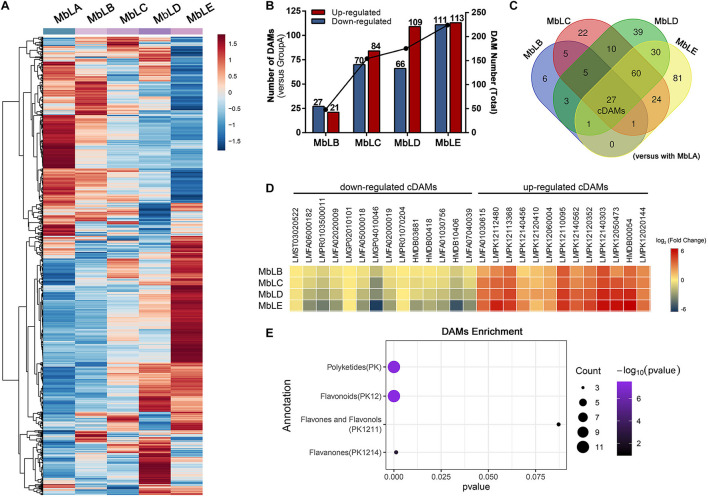
Differential metabolite accumulation in the mulberry leaves at different periods. **(A)** Heat map of all metabolites for MbLA to MbLE. **(B)** The changes of the significantly differentially accumulated metabolites (DAMs) at different periods. **(C)** Venn diagram for all DAMs in MbLB, MbLC, MbLD, and MbLE, with MbLA as the control group. cDAMs refers to common differential accumulation metabolites. **(D)** Heat map of the cDAMs in MbLB, MbLC, MbLD, and MbLE. **(E)** Metabolites annotation enrichment of upregulated cDAMs. The number of DAMs is represented by the circle size.

To explore changes of the metabolites in mulberry leaves under cold stress, the base peak intensity (BPI) chromatogram of mulberry leaves extract was analyzed by UPLC-Q-TOF-MS in positive and negative ion modes (Figure 1B). Efficient metabolomics data processing was performed by Progenesis QI, while pattern-recognition chemometrics was applied for species classification and potential markers discovery (Wang et al., 2014). The differences between samples in different groups were reflected by the PCA score charts (Supplementary Figure 2A). The points of the QC samples in this experiment were closely clustered together, indicating that the whole experimental process had good repeatability and there was no abnormal situation in the data (Supplementary Figure 3). The final statistics showed that 18,598 and 10,239 metabolites were obtained by the positive and negative modes, respectively, 9,016 and 4,699 of which were annotated. With the temperature decreasing, there were more differences of metabolites among groups, which were also verified by the heat map (Figure 2A). In the OPLS-DA score plots, four groups were divided according to different periods clustered with MbLA (Figure 1C). MbLA and MbLB groups exhibited clear separation in OPLS-DA score plots with satisfactory goodness of fit (*R*^2^ = 0.98; *Q*^2^ = 0.9) (Supplementary Figure 2). The two-values associated with fold changes of metabolites before and after the Benjamini-Hochberg method indicating significantly altered metabolites in leaf extracts, which were listed in Table 1. With the temperature decreasing, the number of differential metabolites increased (Figure 2B). We compared all the differential metabolites in mulberry leaves at four temperatures and obtained a total of 27 common differential metabolites (cDAMs; Figure 2C). The heat map of cDAMs showed that from all the upregulation, cDAMs had obvious accumulations and change trends (Figure 2D). mDAMs were annotated and enriched, and 11 compounds were annotated as flavonoids (Figure 2E). From the comparison of upregulated cDAMs, almost all compounds were accumulated while the temperature decreased. These results laid the foundation for our subsequent validation.

**TABLE 1:** Identified the main common differential accumulative metabolites with fold changes in MbLA, MbLB, MbLC, MbLD, and MbLE groups in their *p*-values of mulberry leaves.

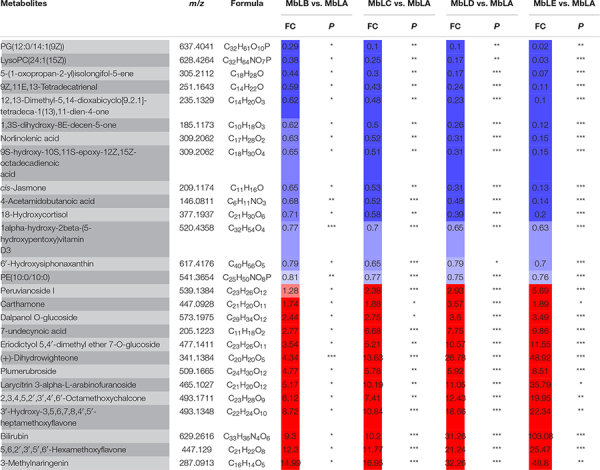

**P*-value: *p < 0.05, **p < 0.01, and ***p < 0.001. Color coded according to fold change using color 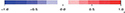.*

### Sequencing and Functional Annotation

In this study, the metabolomics analysis indicated that flavonoids were important differential metabolites in mulberry leaves under different periods. This difference might be regulated by temperature-related genes. To further explore the mechanism of flavonoid enrichment, transcriptomic analysis was performed to identify key genes involved in flavonoid biosynthesis in mulberry leaves. As a result, high quality of data with the average percentage of Q30 bases above 94.24% was obtained, and its comparative efficiency ranged from 71.45 to 77.32%. As we can see from the statistics of sequencing data listed in Supplementary Tables 1, 2, the total clean data reached 103.13 GB with an average clean data of each sample of 5.98 GB. The BLAST was used to perform sequence alignment with RefSeq non-redundant proteins, Swiss-Prot, GO, COG, KOG, PFAM, and KEGG databases to functional annotation. Eventually, a total of 22,939 unigenes containing 1,695 new genes were functional annotations. Additionally, the differentially expressed genes were also identified according to their expression levels in different samples (Supplementary Figure 4). The mapped reads were pieced with StringTie software and compared with the original genetic annotation information to supplement and improve the original genetic annotation information. In addition, several analyses were also performed, including alternative splicing prediction analysis, gene structure optimization analysis, and discovery of new genes.

### Differential Expression of Genes Among Mulberry Leaves Under Different Periods

**FIGURE 3:**
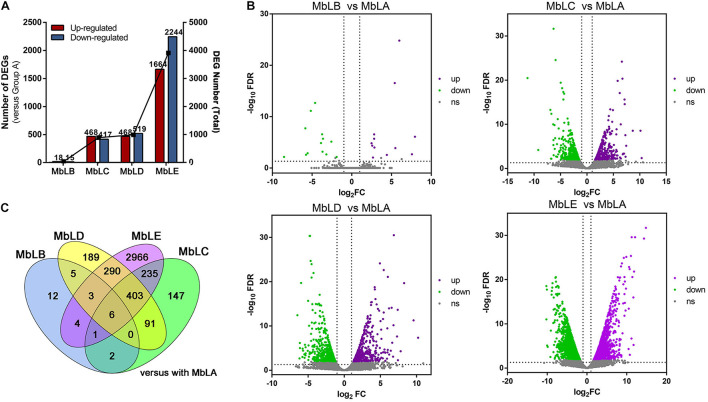
Analysis of the differentially expressed genes (DEGs) at different periods. **(A)** The changes of upregulated DEGs and downregulated DEGs in different groups, with MbLA as control groups. **(B)** Volcano plots for DEGs in the MbLB vs. MbLA, MbLC vs. MbLA, MbLD vs. MbLA, and MbLE vs. MbLA groups. **(C)** Venn diagram for all DEGs in MbLB, MbLC, MbLD, and MbLE, with MbLA as the control group.

To select the DEGs in different groups (Supplementary Table 4), | log_2_^Fold Change^| ≥ 2 and false discovery rate (FDR) <0.01 were used as the screening criteria. The conclusion was that both upregulated and downregulated genes increased with the delay of picking time, which was similar to the differential metabolites accumulation. In groups MbLC and MbLD, the number of DEGs compared with MbLB began to increase, and it reached the maximum in the MbLE (Figure 3A). Specifically, there are 33 DEGs in MbLB, 885 DEGs in MbLC, 987 DEGs in MbLD, and 3,908 DEGs in MbLE, compared with MbLA. The statistical significance of the gene expression level differences in mulberry leaves at different periods was also represented in the volcano plots (Figure 3B). Interestingly, despite the number of DEGs increased, the DEGs in different groups seem various. For instance, by comparing the numbers of common differential genes (Figure 3C) among the groups, we found that there were only six common differential genes. They were *gene 21769*, *gene 8596*, *gene 8678*, *new gene 6800*, *new gene 7715*, and *new gene 7831*. The results also indicated that MbLE maybe has enormous DEGs. Then we compared the DEGs from MbLE with MbLA, and 68 downregulated DEGs and 149 upregulated DEGs were found. All these results indicated that the temperature might had an important role in the gene expression of mulberry leaves.

### Analysis of the Differential Expressed Genes in the Flavonoid Synthesis Pathway

**FIGURE 4:**
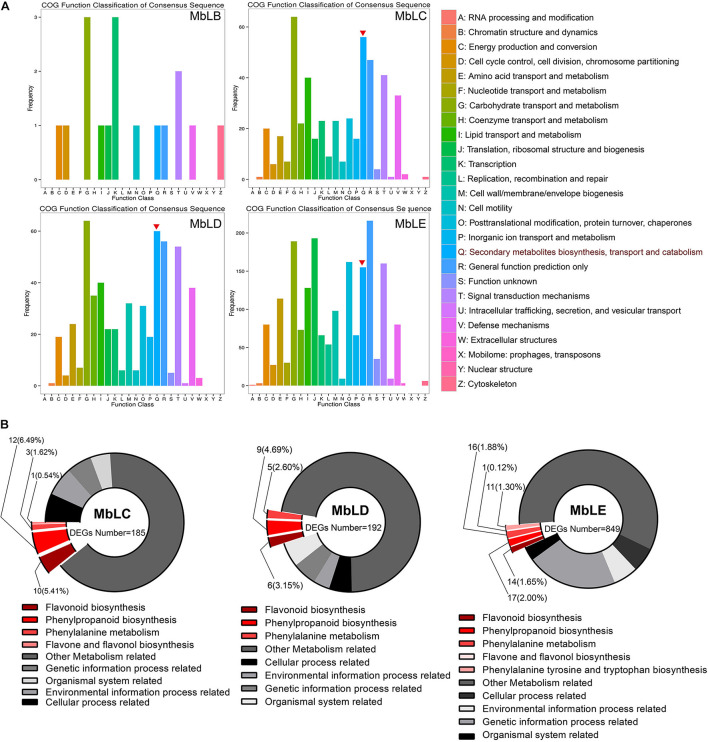
Rich distribution map of differentially expressed genes based on functional annotation. **(A)** COG function classification. The vertical axis represents the gene frequency, and the horizontal axis represents the function class. The red inverted triangles indicated the genes involved in secondary metabolites biosynthesis, transport, and catabolism. **(B)** The enrichment factor represents the ratio of the genes’ proportion annotated to a certain pathway in different genes to the genes’ proportion annotated to this pathway in all genes. The circle’s color represents the *Q*-value, which is the *P*-value after the correction of multiple hypothesis tests. The smaller the *Q* value, the more reliable the enrichment significance of differentially expressed genes in this pathway. The circle’s size indicates the genes’ number enriched in the pathway, and the larger the circle, the more the number of genes.

The recent reports suggested that the main active ingredient of mulberry was flavonoids (Zhang et al., 2018). Hence, we mainly focused on the DEGs involved in flavonoid biosynthesis. To predict individual protein function, we performed COG of protein function classification of the consensus sequence. The annotation of COG indicated that the biosynthetic function of the secondary metabolites of MbLC, MbLD, and MbLE groups was relatively stronger (Figure 4A). KEGG analysis revealed that 26, 20, and 59 genes related to flavonoids synthesis in MbLC, MbLD, and MbLE, respectively. However, the ratio of differential expressed genes decreased owing to the number of flavonoid-related genes increased (Figure 4B).

Enrichment analysis was performed to seek the pathways that involved the DEGs. The pathway enrichment charts (Supplementary Figure 5) show the top 20 pathways with the most reliable enrichment significance (i.e., the lowest *Q*-value). In the top three groups, proteins related to the flavonoid biosynthesis pathway were enriched. For the fourth group, despite such pathways were not in the top 20 pathways with the lowest *Q*-value, there are more metabolic genes were enriched, such as the photosynthesis-antenna pathway. Hence, it was concluded that the flavonoid synthesis in mulberry leaves and the enrichment of their related genes were related to temperature.

### Putative Flavonoid Biosynthesis Pathway in Mulberry Leaves

**FIGURE 5:**
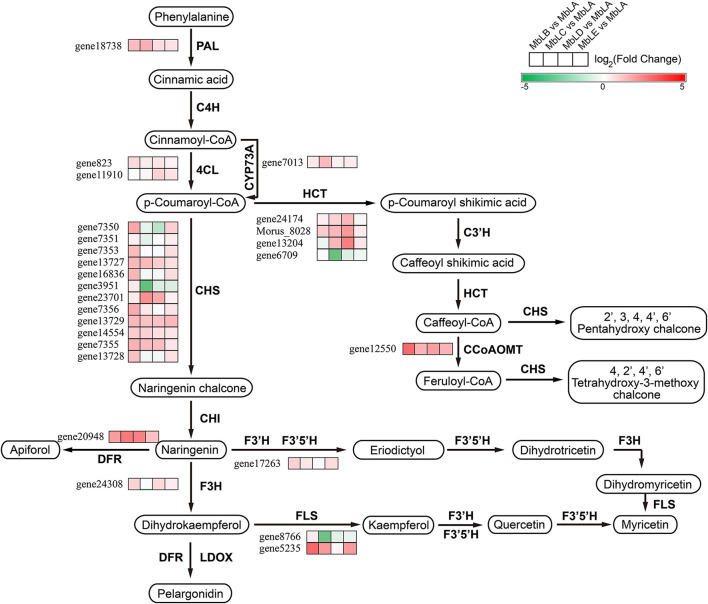
Putative flavonoid biosynthesis pathway in mulberry leaves and gene expression profile of the key enzymes. PAL, Phenylalanine ammonia-lyase; C4H, Cinnamate 4-hydroxylase; 4CL, 4-coumaroyl-CoA ligase; CHS, Chalcone synthase; CHI, Chalcone isomerase; DFR, Dihydroflavonol reductase; F3H, Flavanone 3-hydroxylase; F3′H, Flavonoid 3′-hydroxylase; F3′5′H, Flavonoid 3′5′ hydroxylase; LDOX, leucoanthocyanidin dioxygenase; FLS, Flavonol synthase; HCT, hydroxycinnamoyl transferase.

In organisms, different gene products coordinate with each other to perform related biological functions. Pathway annotation analysis of differentially expressed genes is helpful for further understanding gene function. Combined with the KEGG pathway annotation, we compared and displayed these different genes screened above in the pathway (Figure 5). From Figure 5, we could see that the expression of genes related to flavonoid synthesis is highly correlated with temperature. For example, compared with the expression level of chalcone synthase *gene* 7,356 in MbLE and MbLA, the value of log_2_^Fold^ ^Change^ was 4, indicating that the expression level of this gene was significantly different in the two groups of the mulberry leaves. Additionally, compared the expression level of 4-coumaroyl-CoA ligase gene 11,910 in MbLD and MbLA, log_2_^Fold^ ^Change^ value was 2, indicating that the expression level of this gene in the mulberry leaves of the two groups was also different to some extent. As can be seen from the figure, the number of genes encoding key enzymes of flavonoid synthesis is large, and the expression levels significantly vary at different periods (Supplementary Table 5). It was concluded that the flavonoid synthesis in mulberry leaves and the enrichment of their related genes were related to temperature. Interestingly, expression patterns do not vary in one direction as compared to metabolites and it may due to the insufficient transcription factors, since the process from gene to protein expression is complex. In addition, we analyzed the very top and bottom of our list of the DEGs, and we found that the bottom of the list is a cytochrome P450 71D9-like which is involved in secondary metabolites biosynthesis, transport, and catabolism, while the bottom of the list is a zinc finger BED domain-containing protein involved in replication, recombination, and repair (Kajikawa et al., 2004; Dai et al., 2015; AbuZayed et al., 2019; Wang et al., 2021). This indicated that at an extremely low temperature, mulberry needs to fight against the hostile environment and at the same time, the biosynthesis process of metabolites may be blocked. These results partly agree with the data in Figure 5 and Supplementary Figure 4, in which the gene expression level of flavonoid-related genes was decreased. All the new findings had been added in the article which may provide new evidence in the conclusion that temperature exerted a great influence on the flavonoid biosynthesis in mulberry leaves.

## Discussion

Mulberry leaf is an excellent source of nutrients and phytochemicals. In this study, we explored the changes in relevant compounds and their respective genes at different harvest times through metabolic profiling and transcriptome analyses. The results show that mulberry leaves at different harvest times expressed different flavonoids biosynthesis genes, which results in increasing amounts of flavonoids.

Gene expression associated with flavonoid synthesis was higher at lower temperatures according to the results. However, according to the comparison of the 20 pathways with the highest enrichment significance, we found fewer differentially expressed genes associated with the flavonoid synthesis pathway at the lowest temperature (Supplementary Figure 6). This suggests that genes affecting flavonoid synthesis may not be present in the DEGs lists, or that our criteria for DEGs were too strict. If lower down the criteria, genes affecting flavonoid synthesis may be present in the DEGs lists. However, it is known that when the criteria are lower, the DEGs may become more and more. In order to make the criteria unification, we used the same criteria for the four groups. It is also possible that the plants have higher metabolic demands at low temperatures. For example, the study Guy et al. (1992) studied the influence of growth temperature on the free sugar and sucrose phosphate synthase content of spinach (*Spinacia oleracea*) leaf tissue. They found that the enzyme levels involved in sugar synthesis increased at lower temperatures. In this case, although the expression of genes involved in the flavonoid biosynthesis increased, their proportion decreased. Based on this, we infer that there may be a suitable temperature range for mulberry leaves that will have the greatest effect. The effects of different environmental temperatures on the entire mulberry tree should also be considered.

This study could also be used to verify previous studies on the changes in the levels of flavonoid biosynthesis enzymes and the increased expression of related genes after the weather got cold. We knew that there are many important enzymes affecting flavonoid content from previous studies, such as PAL, CHS, and UDP glucose flavonoid-3-o-glycosyltransferase (UFGT; Qian et al., 2012). Compared with previous studies, we found more genes related to certain key enzymes in flavonoid synthesis. Also, we are inclined to show the rule of metabolites in mulberry leaves with temperature changes and study-related genes on this basis, making the conclusion more convincing.

Additionally, using the annotation information of all genes, we found that certain genes related to the flavonoid biosynthesis pathway were annotated as *iron ion binding* in GO annotation and “flavonoid biosynthesis” in KEGG annotation, such as *Gene 17264, Gene 5235, Gene 5234, Gene 8766, Gene 17263*, and *Gene 5236*. Earlier studies have shown that flavonoids can bind to metal ions and have special effects because of the conjugation and spatial configuration (De Souza and De Giovani, 2004). Based on this, we speculated that enzymes related to flavonoid synthesis have metal ion binding ability, which could be proven through gene annotation. De Souza and De Giovani (2004) found that complexes of metal ions with quercetin, rutin, galangin, and catechin had antioxidant properties. To a certain extent, this confirmed the relationship between flavonoids and metal ions and could invite future investigation.

If we focused on the genes involved in flavonoid synthesis, we could find that genes encoding flavonol synthase (FLS), hydroxycinnamoyl transferase (HCT), dihydroflavonol reductase (DFR), catechol-*O*-methyltransferase (COMT), and CHS showed a relatively higher difference. On the other hand, by analyzing the pathways, we found that the pathways with the greatest degree of gene change were those related to plant metabolism. The synthesis pathway of flavonoids also increased with temperature decrease trend. However, in MbLE, the synthesis pathway enrichment of flavonoids was not in the top 20, the main enrichment pathways were photosynthesis, respiration, amino acid synthesis pathway, and amino acid synthesis pathway. It was possible that genes related to flavonoid synthesis were still enriched and upregulated, but the changes were smaller than those in other basic metabolic pathways.

In summary, we investigated the metabolomics and transcriptome of mulberry leaves and compared DEGs and DAMs at different periods. Using the functional group analysis of GO and KEGG pathway annotations, compounds and genes related to the flavonoid biosynthesis pathway at different periods were identified and used to speculate that lower temperatures induce the expression of flavonoid-related genes. We also compared and displayed the screened differential genes in the pathways as a preliminary exploration of the biosynthesis pathway of the flavonoids in mulberry leaves, which requires further study. Finally, our study could serve as a reference for the analysis of metabolomic and transcriptomic data from other medicinal plants.

## Data Availability Statement

The datasets presented in this study can be found in online repositories. The names of the repository/repositories and accession number(s) can be found below: NCBI SRA BioProject, accession no: PRJNA533997.

## Author Contributions

D-QX, S-YC, Y-CZ, and Y-PT conceived the experiments and helped to coordinate support and funding. D-QX, J-QZ, S-YC, S-JY, and MT performed the research, drafted, and revised the manuscript. J-QZ, H-FL, and Y-YC participated in the experiments. D-QX and Y-CZ analyzed the data and edited the manuscript. All authors read and approved the final manuscript.

## Conflict of Interest

The authors declare that the research was conducted in the absence of any commercial or financial relationships that could be construed as a potential conflict of interest.

## Publisher’s Note

All claims expressed in this article are solely those of the authors and do not necessarily represent those of their affiliated organizations, or those of the publisher, the editors and the reviewers. Any product that may be evaluated in this article, or claim that may be made by its manufacturer, is not guaranteed or endorsed by the publisher.

## Footnotes

1
http://trinityrnaseq.sf.net

2
http://www.geneontology.org/

3
http://www.ncbi.nlm.nih.gov/COG/
