# Supplementary material for: Morus alba L. Leaves – Integration of Their Transcriptome and Metabolomics Dataset: Investigating Potential Genes Involved in Flavonoid Biosynthesis at Different Harvest Times
Source: Front Plant Sci. 2021 Nov 16;12:736332. doi: 10.3389/fpls.2021.736332 (PMC8637763; doi:10.3389/fpls.2021.736332)
Supplement: Supplementary file 1 [file Presentation_1.pdf]

Supporting information

Morus alba L. **Leaves - Integration of their Transcriptome and Metabolomics Dataset: Investigating Potential Genes Involved in Flavonoid Biosynthesis at Different Harvest Times**

Ding-Qiao Xu[#1], Shu-Yan Cheng[#2], Jun-Qing Zhang[3], Han-Feng Lin[2], Yan-Yan Chen[1], Shi-Jun Yue[1], Meng Tian[1], Yu-Ping Tang*[1], Yu-Cheng Zhao*[2]

[1] Key Laboratory of Shaanxi Administration of Traditional Chinese Medicine for TCM Compatibility, School of Pharmacy, Shaanxi University of Chinese Medicine, Xi'an 712046, Shaanxi, China.

[2] Department of Resources Science of Traditional Chinese Medicines and State Key Laboratory of Natural Medicines, School of Traditional Chinese Pharmacy, China Pharmaceutical University, Nanjing 210009, Jiangsu, China

[3] Department of Metabolism, Digestion & Reproduction, Faculty of Medicine, Imperial College London, South Kensington Campus, London SW7 2AZ, U.K.

[#] These authors have contributed equally to this work.

Correspondence:
Yu-Ping Tang
yupingtang@sntcm.edu.cn

Yu-Cheng Zhao
zhaoyucheng1986@126.com

Chemicals and reagents

All the solvents and reagents used in this study were of LC or LC-MS grade. LC-MS grade acetonitrile and methanol were purchased from Merck (Darmstadt, Germany). Formic acid was purchased from Sigma-Aldrich (St. Louis, MO).Internal standard 2-chloro-l-phenylalanine (CAS NO: 103616-89-3) was obtained from Aladdin (Shanghai, China). IS was added either before or after the extraction to all samples, blanks, and quality control samples. Methodological blanks were extracted and analyzed along with each sample set. To evaluate the effect of obscuring variation due to different matrix strengths and analytical drift, pooled samples were run at both full and half concentration (diluted with menthol) at least three times throughout a sample set. The final concentration of IS is 0.3 mg / mL. Deionized water was prepared by a Milli-Q water purification system (Millipore, France).

Table S1 Statistical results of functional annotation of new genes in mulberry leaves

Item	Data
Total clean raw reads (GB)	103.13
Total nucleotides (bp)	93,282,183
GC percentage	37.41%
Average read length (bp)	4,025
Total scaffolds	23,174
Length range (bp)	171 - 229,817
N50 (bp)	5,499
NR	1,690 new genes
Swiss-Prot	962 new genes
KEGG	544 new genes
COG	396 new genes
GO	1,097 new genes

Table S2 The statistics of sequencing data of each mulberry leaves

#Sample ID	Read Sum	Base Sum	GC (%)	Q20 (%)	Q30 (%)
T1-1	20001700	5.98E+09	45.01	97.71	93.66
T1-2	20906907	6.23E+09	46.12	98.12	94.48
T1-3	23647256	7.03E+09	46.09	98.14	94.44
T2-1	20766588	6.19E+09	45.57	98.00	94.18
T2-2	23206694	6.90E+09	45.98	98.08	94.27
T2-3	20130987	6.00E+09	45.72	98.01	94.12
T3-1	24540843	7.33E+09	45.8	97.95	94.06
T3-2	22824010	6.81E+09	46.05	97.97	94.10
T3-3	28029717	8.35E+09	45.76	98.19	94.56
T4-1	22916901	6.83E+09	45.65	98.11	94.39
T4-2	21771590	6.50E+09	45.77	98.04	94.25
T4-3	23857077	7.11E+09	45.86	98.04	94.17
T5-1	21792654	6.49E+09	45.67	98.12	94.46
T5-2	21258363	6.32E+09	46.32	98.09	94.36
T5-3	30375691	9.06E+09	45.75	98.02	94.12

Table S3 The statistics of RNA quality of each mulberry leaves

Samples	RIN value	28S/18S	OD260/280	OD260/230
T1-1	7.9	2.01	2.13	1.20
T1-2	7.6	2.54	2.13	0.65
T1-3	7.6	1.30	2.99	1.34
T2-1	7.9	1.89	2.15	0.45
T2-2	7.8	1.80	2.14	1.74
T2-3	8.0	2.45	2.14	0.82
T3-1	8.0	1.90	2.13	0.26
T3-2	7.8	1.60	2.12	2.20
T3-3	8.1	2.22	2.13	0.78
T4-1	8.3	1.88	2.13	2.20
T4-2	8.0	2.13	2.14	0.53
T4-3	8.3	1.96	2.14	0.84
T5-1	8.0	1.40	1.98	0.61
T5-2	7.8	2.09	2.15	0.69
T5-3	7.8	1.90	2.16	0.24

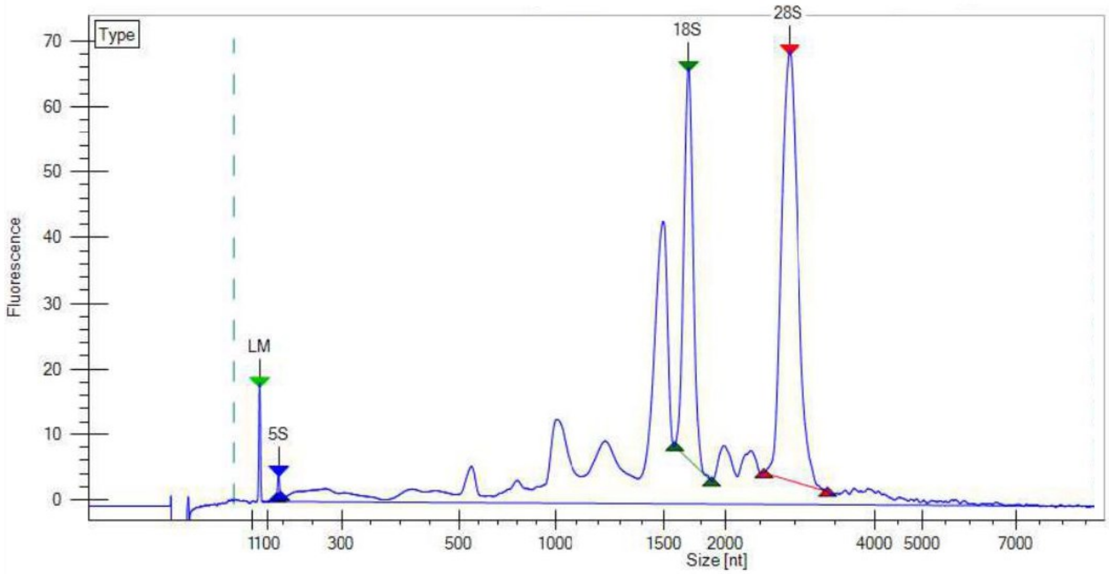

Figure S1 The quantity and quality of RNA were determined by Agilent 2100 Bioanalyzer system. For the computation or the RNA integrity number, the electropherogram is partitioned into regions as shown.

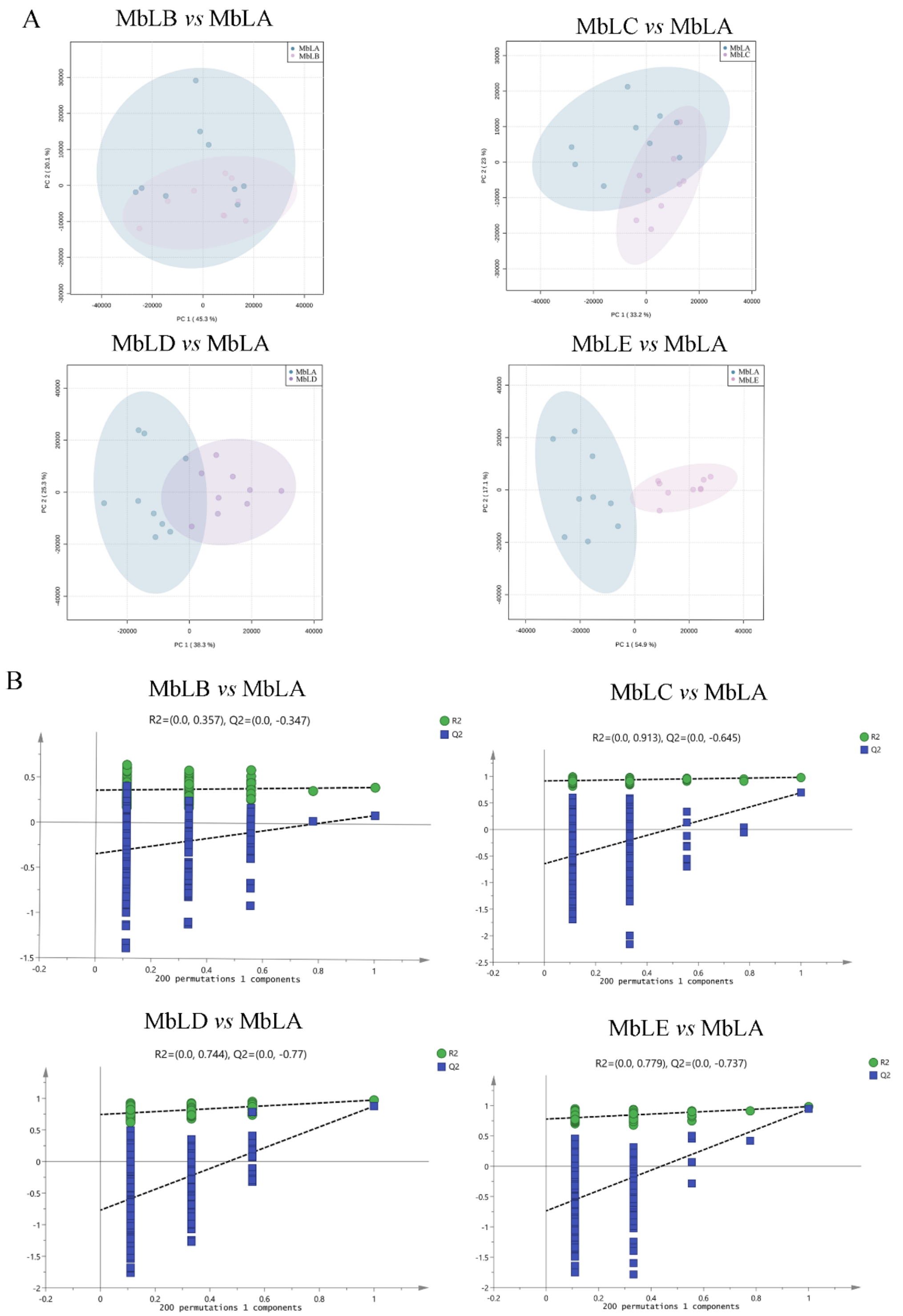

Figure S2 Score plots analysis based on UPLC-QTOF-MS data. (A) PCA score plot of metabolite profiles from MbLB to MbLE, with MbLA as the control group. Each point represents an independent biological replicate, and each ellipse represents the 95% confidence interval. (B) The OPLS-DA scatter plot from mulberry leaves extracts of statistical validation obtained by 2000 times permutation test, with R^2 and Q^2 values in the vertical axis, the correlation coefficient (between the permuted and true class) in the horizontal axis, and the ordinary least squares (OLS) line for the regression of R^2 and Q^2 on the correlation coefficients.

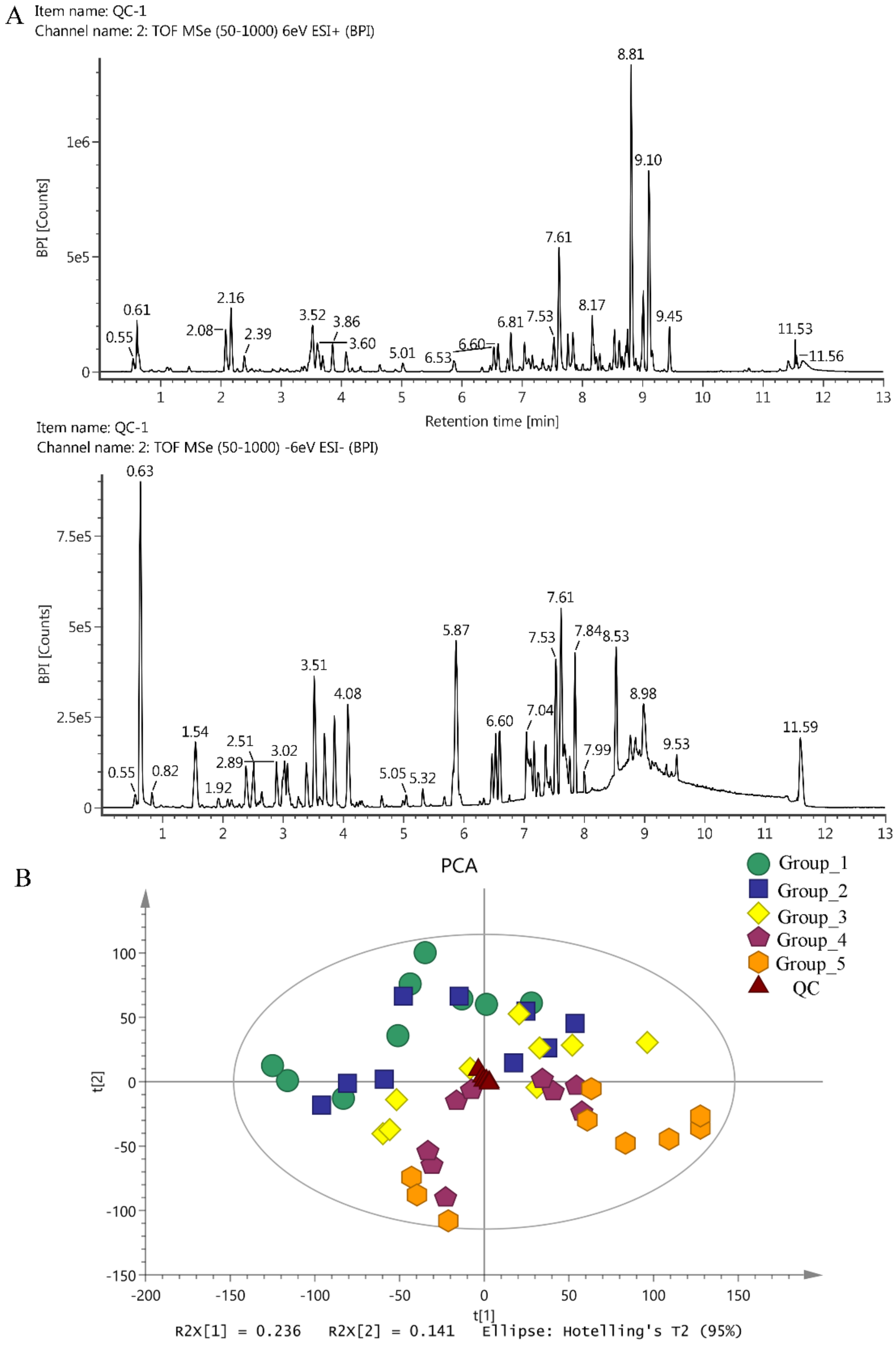

Figure S3 Analysis of compounds extracted from QC sample of mulberry leaves using UPLC-QTOF/MS. (A) Base peak intensity (BPI) chromatogram of mulberry leaves extract analyzed by UPLC-QTOF-MS in positive and negative ion mode. (B) Principal component analysis score chart for all samples. Each colored dot represents a sample, and the distribution and dispersion of the points indicate the differences between samples to some extent. The points of the QC samples in this experiment were closely clustered together, indicating that the whole experimental process had good repeatability and there was no abnormal situation in the data.

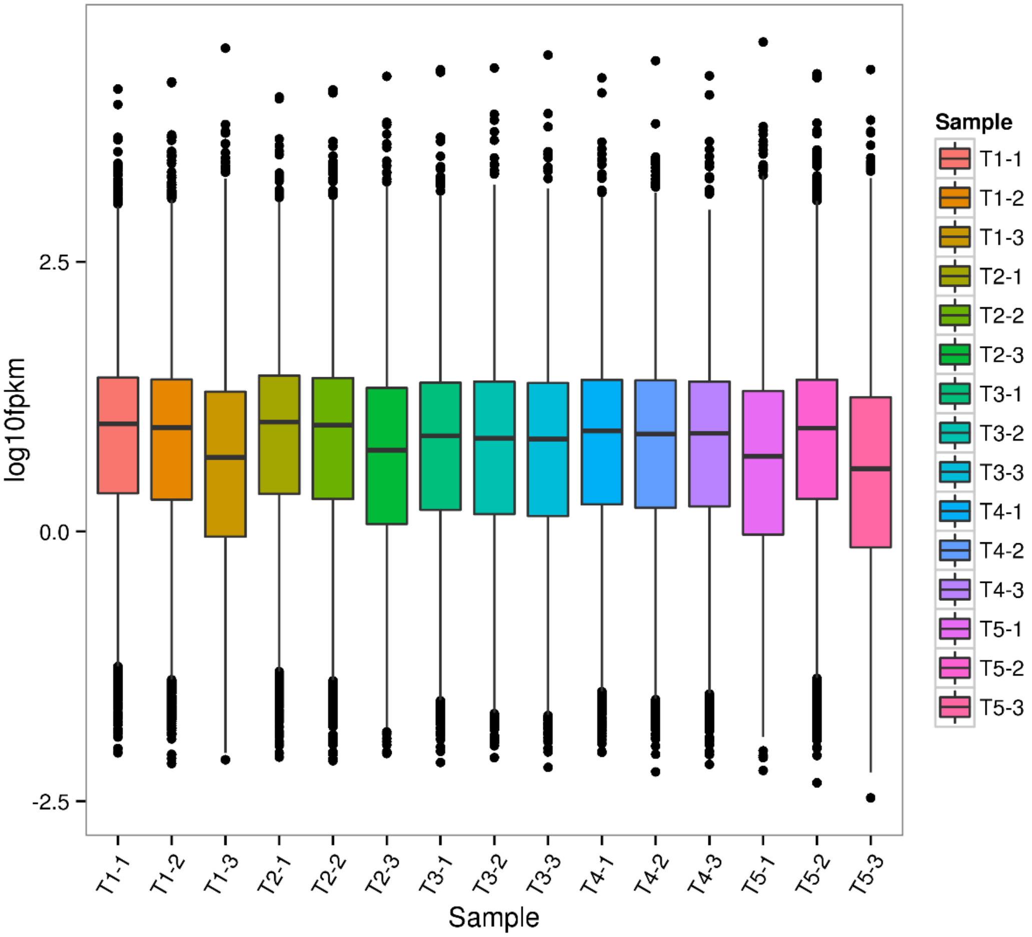

Figure S4. The FPKM carton diagram of each sample. The abscissa represent different samples. The ordinate represents the log value of sample expression quantity FPKM. The expression level of each sample from the perspective of total dispersion of expression quantity.

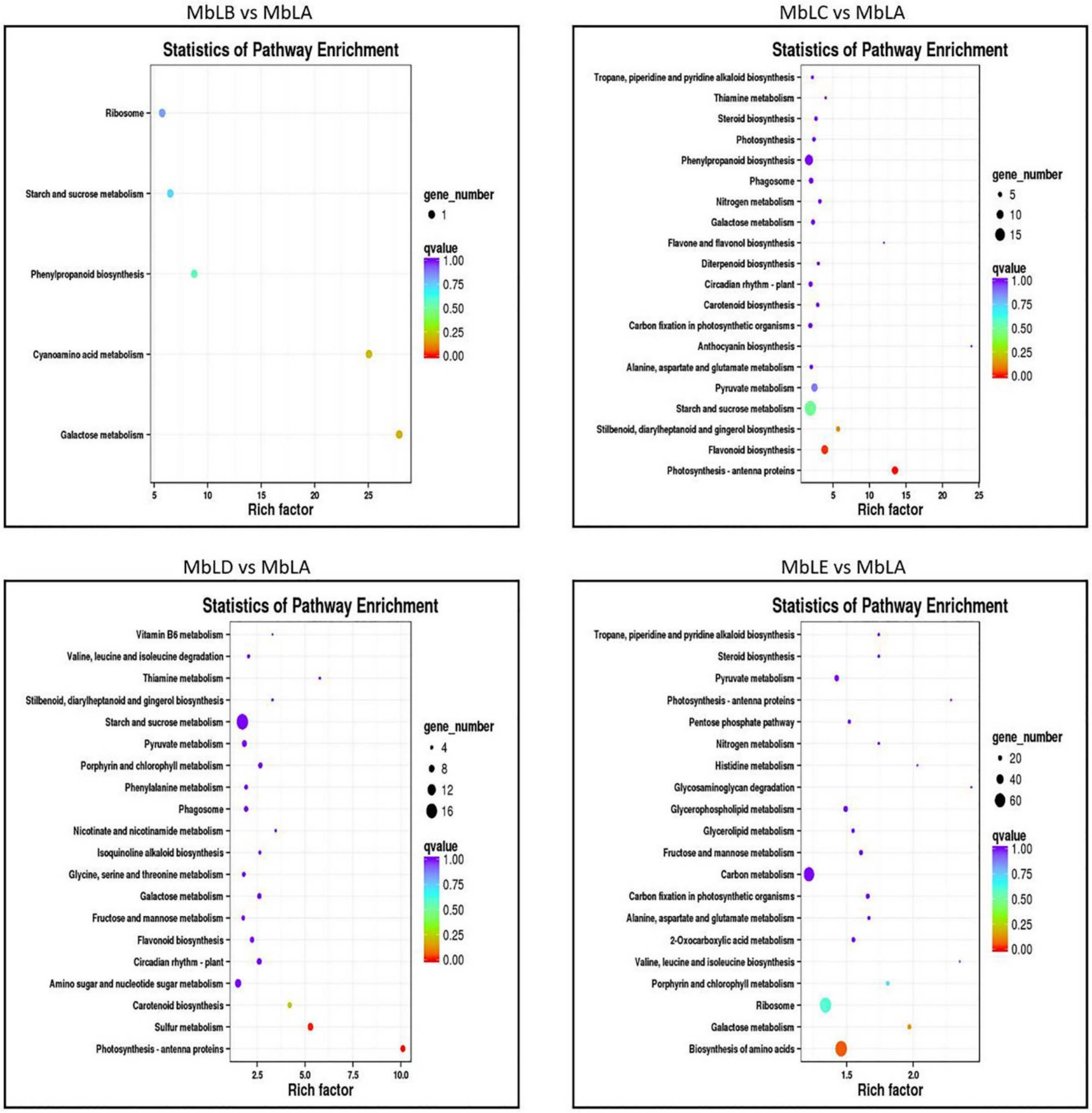

Figure S5. Functional classification and annotation analysis of the differentially expressed genes (DEGs). (A) COG function classification of consensus sequences in MbLB, MbLC, MbLD, and MbLE. (B) Pie chart showing the Kyoto Encyclopedia of Genes and Genomes (KEGG) functional annotation distribution of all the DEGs in MbLC, MbLD, and MbLE groups. The exploded slices of the pie represent the functional pathways related to flavonoid biosynthesis pathways. The numbers in the pie chart indicate the number of DEGs annotated to a certain pathway and the ratio of the genes' number that are annotated to a certain pathway and the genes' number that are annotated in total.

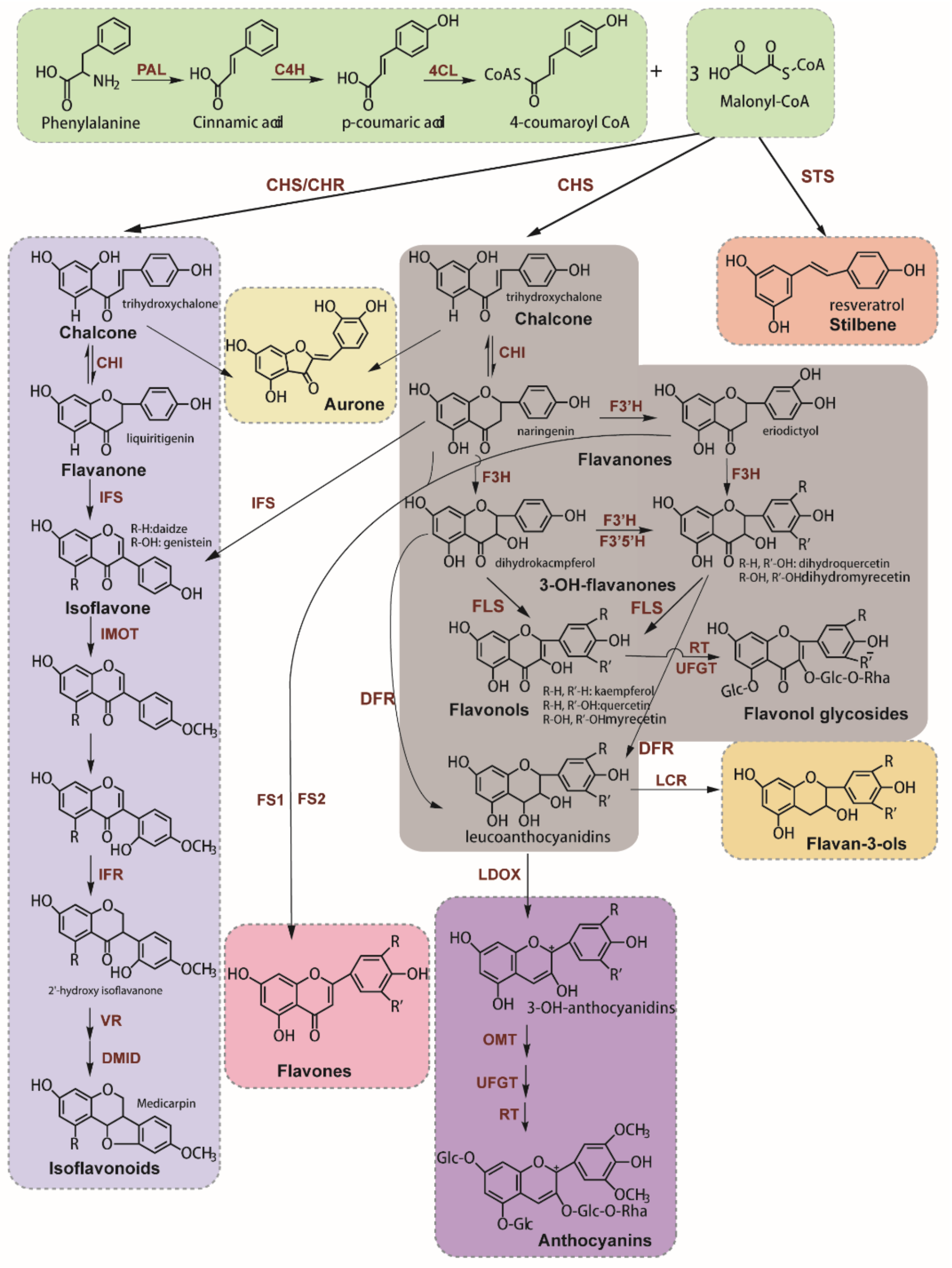

Figure S6 Putative flavonoid biosynthesis pathway in mulberry leaves.
